# Nano-Enriched Self-Powered Wireless Body Area Network for Sustainable Health Monitoring Services

**DOI:** 10.3390/s23052633

**Published:** 2023-02-27

**Authors:** Bassem Mokhtar, Ishac Kandas, Mohammed Gamal, Nada Omran, Ahmed H. Hassanin, Nader Shehata

**Affiliations:** 1 College of Information Technology, United Arab Emirates University, Abu Dhabi 15551, United Arab Emirates; 2 Department of Electrical Engineering, Faculty of Engineering, Alexandria University, Alexandria 21544, Egypt; 3 Center of Smart Materials, Nanotechnology and Photonics (CSMNP), Smart CI Research Center, Alexandria University, Alexandria 21544, Egypt; 4 Department of Engineering Mathematics and Physics, Faculty of Engineering, Alexandria University, Alexandria 21544, Egypt; 5 Department of Textile Engineering, Faculty of Engineering, Alexandria University, Alexandria 21544, Egypt; 6 Wilson College of Textiles, NC State University, Raleigh, NC 27606, USA; 7 Kuwait College of Science and Technology (KCST), Doha Superior Rd, Jahraa 13133, Kuwait; 8 USTAR Bioinnovations Center, Faculty of Science, Utah State University, Logan, UT 84341, USA

**Keywords:** wireless body area networks, bio-nanosensors, nano-materials, energy harvesting, flexible electronics

## Abstract

Advances in nanotechnology have enabled the creation of novel materials with specific electrical and physical characteristics. This leads to a significant development in the industry of electronics that can be applied in various fields. In this paper, we propose a fabrication of nanotechnology-based materials that can be used to design stretchy piezoelectric nanofibers for energy harvesting to power connected bio-nanosensors in a Wireless Body Area Network (WBAN). The bio-nanosensors are powered based on harvested energy from mechanical movements of the body, specifically the arms, joints, and heartbeats. A suite of these nano-enriched bio-nanosensors can be used to form microgrids for a self-powered wireless body area network (SpWBAN), which can be used in various sustainable health monitoring services. A system model for an SpWBAN with an energy harvesting-based medium access control protocol is presented and analyzed based on fabricated nanofibers with specific characteristics. The simulation results show that the SpWBAN outperforms and has a longer lifetime than contemporary WBAN system designs without self-powering capability.

## 1. Introduction

Wireless Body Area Network (WBAN) is a developing networking and communication technology that is applied for health monitoring [1]. WBAN integrates sensors with actuators on or within the human body to monitor and measure biometrics. It works over a short-range wireless communication protocol to support data transfer between sensors and a collector and is sent wirelessly to a device outside the human body. WBAN is currently involved in various applications and services related to health, such as remote patient monitoring, patient rehabilitation, biofeedback, and assisted living. Moreover, WBAN can be used in non-medical applications, such as fitness and performance monitoring and biometric signatures. In addition, different technologies are deployed to enable sustainable WBAN ranging from sensing, and communication, to management.

Wearable biosensors can be integrated with WBAN and use various materials, such as clothes or bandages to help in providing continuous monitoring of human health [2]. One major challenge in using such sensors and related devices is identifying a reliable energy supply that can be used as most of these sensors depend on finite capacity rechargeable batteries. Many research trials and efforts have focused on addressing energy resource constraints by enabling energy-efficient communication protocols and designing effective WBAN topologies. For example, due to the varying energy generated by the human body, a nanogenerator was presented in [3] that enables electricity generation based on transforming the mechanical energy generated in the body by exploiting the piezoelectric effect in some materials [4].

Some approaches have addressed the limited energy constraints of sensors used in WBAN by providing techniques for battery recharging whether using wireless power transfer or energy harvesting. One approach to tackle the reliable energy supply challenge is considering new technologies to develop novel materials supporting energy harvesting processes for miniaturized sensor devices. Nanotechnology is the science that deals with understanding, investigating, and fabricating materials, called nanomaterials. Their dimensions are at the level of nanometers, which is important for determining specific characteristics of the materials [5]. These nanomaterials acquire certain physical, chemical, mechanical, and electrical characteristics that make them suitable a set of applications in different fields, such as health monitoring and energy harvesting. Self-powered energy sources have been extensively studied over the last two decades, including piezoelectric bulk, micro, and nanomaterials for different applications of energy harvesting, wearable electronics, and industrial transducers. Different fabrication technologies of energy harvesting units have been studied in the literature, with the aim of generating thin flexible films that are lightweight and biocompatible/biodegradable [6]. According to the concept of a higher surface-to-volume ratio, nanofibers membranes can be considered the optimum polymeric piezoelectric thin film materials [7,8,9]. Flexible nanofiber mats with a diameter range between tens of nanometers to several micrometers are fabricated through different mechanisms such as electrospinning, wet-spinning, and solution-blown spinning [10,11]. Among the various polymers used in the field of piezoelectric mats, poly(vinylidene fluoride) (PVDF) is considered one of the most promising materials due to the fluorine atoms’ electronegativity and consequently the electric dipole formation between hydrogen and fluorine along with the polar crystalline structure nature of PVDF [12,13]. Inside PVDF, there are two phases of the formed dipoles. They are the main polar phases of β-phase, and are responsible for the piezoelectric behavior of the PVDF [14,15] The non-polar phase of α-phase can be transformed to β-phase through mechanical stretching, and electrical poling, thereby improving the piezoelectric response of the mat [16,17].

Flexible electronics and bio-electronics are emerging electronics fields that provide integrated systems and solutions for reliable and seamless contact for monitoring health and many activities of the human body [18,19]. WBAN exploit such technologies to offer various services. However, there are some challenges that should be tackled in order to apply such systems and solutions efficiently. The main challenge is in providing an energy supply to enable reliable connection of wearable biosensors in a wireless environment affected by interference from different devices and to provide time-sensitive services. A lack of a reliable solutions to this challenge significantly affects the lifetime and reliability of any WBAN implementing these technologies.

In this work, we provide feasible solutions for mitigating this energy supply-related challenge. We exploit nanotechnology to fabricate piezoelectric nanomaterials with specific electrical and physical characteristics, and consequently design nano-biosensors in the form of wearable piezoelectric mats of flexible bio-electronic devices. The targeted mats are fabricated using synthesized poly(vinylidene fluoride) (PVDF) solution and turned into nanofibers mats via an electrospinning technique. The electric field of the electrospinning process supports the alignment of electric dipoles inside the organic material, which is the main physical feature required for a piezoelectric response. The synthesized nanofibers mats can be powered via the embedded materials by the movement of some parts of the human body, such as joints, and generated cardiac signals. Then, a design for a sustainable WBAN is proposed which employs an energy harvesting-based MAC protocol for health monitoring applications. This WBAN consists of self-powered nano-biosensors distributed over the human body on the skin or on clothing to monitor and read vital health-related parameters. Each suite of nano-biosensors forms a microgrid where power is managed for them based on controllers driven by machine learning systems to ensure the suitable operation of the sensors.

Our main contributions in this work are as follows: We propose (a) a system model and design for a self-powered WBAN employing an energy harvesting-based MAC protocol with built-in piezoelectric nano-biosensors forming a sensory system and microgrids that support power to other sensors in the network; and (b) a novel design for low-cost piezoelectric nano-biosensors that can read and generate data about body health status and that are manufactured using nano-enriched materials with specific characteristics for energy harvesting and self-powering. The designed WBAN can be applied and evaluated over various health monitoring services which are used in telemedicine and chronic disease monitoring.

This paper is organized as follows: Section 2 discusses the related work from the literature along with our new contribution. The system model, communication topology, and management architecture of self-powered WBAN care are discussed in Section 3. Section 4 presents the fabrication and characterization of piezoelectric nano-enriched materials. The self-powered WBAN is evaluated in Section 5. Then, the paper concludes in Section 6 by highlighting some future directions for this work.

## 2. Literature Review

The research on integrating WBAN with telemedicine and health monitoring applications started a long time ago [1]. This integration depends on the Internet core and data analytics to generate real-time guidance and feedback to users in many areas. WBAN depend on utilizing distributed sensors throughout the human body with various placement strategies, on or under the skin, to monitor health-related parameters to improve the quality of life. To extend WBAN efficacy, different communication infrastructures can be applied from 4G/5G technologies to wireless networking and satellite infrastructure [2]. The adoption of WBAN in healthcare-related applications requires continuous operation and monitoring. Thus, a high rate of energy consumption and limitation on the network lifetime is expected. Consequently, there are challenges and criticality in providing long-term efficient monitoring and service provisioning. In the literature, diverse solutions have been developed to tackle those challenges. Some of these solutions that are discussed enable long-term operations and extend the lifetime of WBAN. Some methods may depend on generating electrical energy from ambient energy sources [20]. The authors investigated energy-harvesting interface circuits for WBAN.

In [21], the authors presented a MAC protocol to support a radio frequency energy harvesting WBAN. They adopted TDMA-based scheduling to minimize energy consumption due to interference. They also proposed a power adjustment scheme to optimize power transmission and developed a scheme to optimize data throughput to enable reliable data delivery and communications. Other solutions targeted energy harvesting and energy saving in WBAN. For example, an algorithm was developed to find the maximum number of dominating sets and the minimum number of nodes required to relay data delivery in an energy harvesting WBAN [22]. Another solution developed an energy-efficient harvested-aware clustering and data routing protocol to operate in WBAN [23]. This protocol adopted a dynamic approach to select cluster heads and avoided the transfer of redundant data among nodes to save energy.

The authors in [4] surveyed different nanotechnology-based mechanisms of generating power, namely, piezoelectric nanogenerators (PENG), triboelectric nanogenerators (TENG), and pyroelectric nanogenerators (PYENG). These mechanisms require the fabrication of materials with specific characteristics: they must be lightweight and flexible, and have good electrical performance. Accordingly, such materials can be developed and exploited as nanogenerators to collect energy from the body and power sensors on wearable electronics. Different types of such sensors can be used as biosensors to create self-powered WBAN to monitor body health-related parameters, such as blood pressure and cardiac signals.

Various energy-harvesting sources of nonelectric renewable energy were investigated in [24,25,26] to overcome the problem of limited energy supplies for sensor devices in WBAN. These sources include biochemical sources, ambient sources, and biomechanical sources. Biochemical sources target electrochemical energy and use various harvesting techniques, such as enzymatic biofuel cells. The ambient sources depend on light or electromagnetic radiation as a source of energy and use photovoltaic cells or thermoelectricity for energy harvesting. The last type is biomechanical sources which depend on vibration energy and use, for instance, piezoelectric materials for energy harvesting. A biomechanical energy source that depends on internal and external motions can provide good levels of energy. In particular, there are various movement sources in the body, such as the heartbeat and muscle movement, that can be used to generate energy and managed by aggregators for effective usage.

Optimization problems are formulated to maximize energy efficiency of energy-harvesting-enabled WBAN [27]. The aim of optimization is to plan how to allocate energy to crucial sensors in WBAN to prolong their lifetime and improve their performance. In [25], the authors surveyed different techniques of energy harvesting for WBAN and optimized approaches for exploiting the harvested energy in WBAN. The optimization depends on addressing protocols for WBAN at the medium access control (MAC), routing, and physical layers. In [28], Changle et. al. presented a model for MAC protocol called LEDA to help extend the WBAN lifetime. The protocol depends on IEEE 802.15.6 and it employs two modes of wireless signal transmission and reception in a shared medium. The first is a multi-beam directional mode with carrier sense multiple access/collision avoidance, and the second is a single-beam directional mode with time division multiple access. The authors presented an analysis of the LEDA operations based on a dynamic polled allocation period to enable reliable data transmission.

## 3. SpWBAN System Model

We propose a SpWBAN architecture that consists of three levels as follows: The first level includes the self-powered nano-biosensors that run relying on materials for energy harvesting. The second level incorporates the communication and management of self-powered nano-biosensors within the SpWBAN topology. The third level includes the management of data collected from different sources, such as various nano-biosensors, other WBAN, and constructed databases for further analysis and actions. Additionally, this level incorporates management of the power generated from the energy harvesting materials associated with biosensors. In the first level, there are wireless self-powered biosensors fabricated with energy harvesting nanomaterials that collect data and forward them to a collecting device. The next section, i.e., Section 4, presents the fabrication of the nano-biosensor based on piezoelectric nano-enriched materials. The second level explains how communication among nano-biosensors is conducted where a topology for SpWBAN is created and various communication technologies, such as Bluetooth and IEEE 802.15.6 protocol, can be used. In the third level, the data gathered at collectors and hubs are sent over 4G/5G technologies to powerful analysis stations over the Internet to be preprocessed and cleaned in order to be used for further quantitative and qualitative mathematical analysis. Moreover, a set of predefined thresholds are declared based on pre-simulated samples to control the data and customize the performed mathematical analysis.

**Figure 1:**
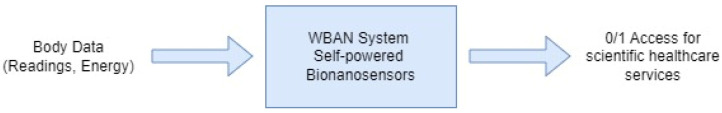
Problem design of the services allocation for patients.

A realistic application for this system model would be the healthcare service allocation for patients by healthcare providers in a region which is equipped with a 4G/5G tower. Using data gathered from people with a WBAN and by defining a set of thresholds based on the predetermined specifications of the 4G/5G tower’s hardware as well as pre-simulated samples, mathematical and machine learning-based analysis can be implemented on the data of the region, thereby producing insights on healthcare service provisioning in the region. Therefore, according to the design problem described in Figure 1, the system is concerned with performing a mathematical analysis taking into consideration the predetermined thresholds, as previously mentioned. This system collects the body information and then produces a permission concerning whether to grant (1) an access or not (0) for each patient.

**A.**  **Data Management and Routing in SpWBAN**

In the second level of management, using machine-learning algorithms and learned features related to the type of required healthcare services, decisions are taken by intelligent systems found on stations in the backhaul Internet separate from the SpWBAN. These decisions are made based on data/energy readings to achieve efficient actions in real-time. This can help in offering future predictions and planning for provisioning healthcare services and also replacing wearable health sensors and devices if they malfunction or are energy depleted.

In addition, the decision can ensure there are reliable routing plans for data in the SpWBAN, among SpWBAN and between SpWBAN and the Internet core. Different routing algorithms can be applied to enable efficient data routing for real-time health monitoring applications [29]. In general, routing with a SpWBAN can be performed on a one-hop basis between sensors and the collecting unit. However, in the case of different levels of communication and management between multiple WBAN and the Internet, multichip routing is applied.

**B.**  **Energy Harvesting-based MAC for SpWBAN**

**Figure 2:**
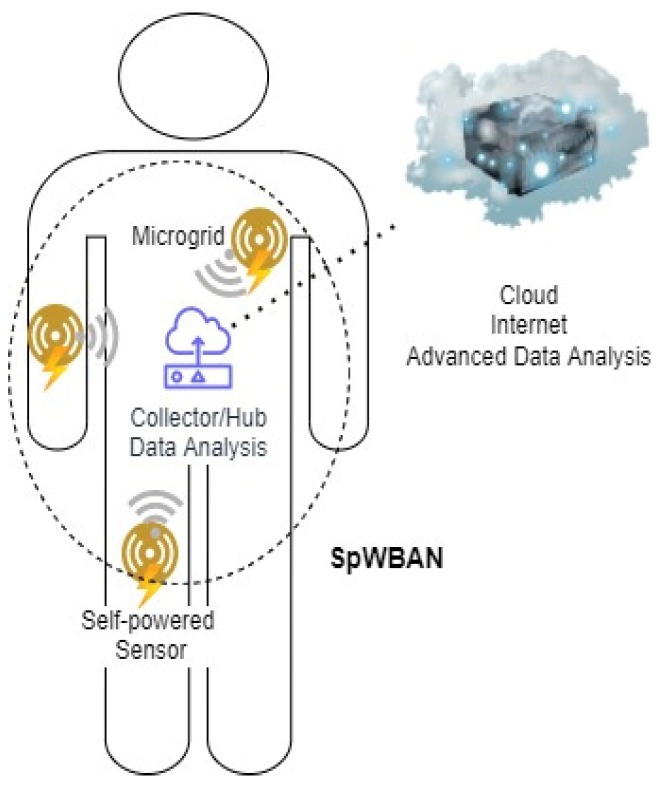
System model of SpWBAN.

Based on the system model presented in Figure 2 below, the WBAN on each person is supported by a microgrid that integrates the energy harvesting units with the sensors to assist in operating the sensors and other devices. Therefore, data about the power/energy levels from these units are collected and analyzed to better use the self-powered wearable devices.

We assume a one-hop star topology for each microgrid where the collector receives readings wirelessly from the sensors. The collector applies some operations related to data analysis on these readings and then forwards them to the cloud for advanced data analysis. We adopt a hierarchical communication network architecture to achieve reliable and managed data transmission between the sensors and the cloud. In addition, when there is more than one microgrid on the body, there is a controller to manage traffic from them.

In each WBAN (i.e., the first management level), there is a collecting device that performs a local analysis and makes decisions before forwarding the data/power readings to powerful units and stations at the SDN-based management level (i.e., the second management level). The SDN-based management depends on a set of devices or controllers that run machine learning models to perform data analysis and to support decisions taken by the SDN controllers.

In this work, we discuss the mathematical model of SpWBAN that employs an energy harvesting-based MAC protocol with one management level that can generate decisions related to the allocation of health monitoring services. There is also the capability for the management level to forward readings and findings to an Internet cloud, i.e., an advanced management level, for further data analysis and processing.

**C.**  **SpWBAN Mathematical Model**

We present a mathematical model for SpWBAN, which is a human body-centered network where a suite of connected various sensors with a variety of traffic. We assume that the MAC protocol at SpWBAN will depend on single-beam and multi-beam directional antennas that can minimize signal interference among transmitting sensors and accordingly minimize energy consumption [28,30].

We adapted the MAC protocol for WBAN presented in [28] and its related antenna model to be used by sensors and the collector, as shown in Figure 2. The implemented MAC protocol enables reliable data transfer between sensors and the collector. The protocol also allows three different time periods as follows: one time period for multi-beam transmission and reception, another time period for single-beam transmission and reception, and the time period for inactivity.

We conducted an analysis of the generated voltage from the applied energy harvesting setup using polymers or piezopolymers and using a piezoelectric organic material such as PVDF. We formulated the generated voltage, as illustrated in the equations in Table 1 where three related varying parameters have effects. We compared the ranges of applied forces and frequencies with related work in the literature [31,32].

In addition, based on the study presented in [31,33], the electrical energy generated from mechanical energy applied to piezoelectric materials due to a specific source can be computed using the equation:

(1) Ep,source=(V2Z)Tg

where E_p,source_ is the electrical energy generated from the piezoelectric material according to a specific source, V is the generated voltage, Z is the equivalent impedance of the piezoelectric material which includes the resistive and capacitive loads, and Tg is the energy generation time. PVDF nanofibers served as the piezoelectric material, which is presented as a circuit model of a resistor connected in parallel to a capacitor.

Based on the adopted models in [28,31,34,35], we assume that the decay of the energy can be be expressed by the following equation where an electrical circuit-based model for energy discharging in the fabricated piezoelectric material was used.

(2) $$E_{t,source}=E_{p,source}e^{-T_{inactive}/RC}$$

Equation (2) computes the amount of energy harvested $E_{t,source}$ to power the biosensor. $T_{inactive}$ represents the period that there is no motion or activity in the human body. *R* is the resistance of conductive wet-spinning on the PVDF nanofibers. *C* represents the equivalent capacitance related to the stiffness of the piezoelectric material. This means that if there is a long time without activity, the sensors will be depleted and out of power.

We propose three different types of energy harvesting sources for the implemented bio-nanosensors in SpWBAN as follows: the heart beat source (S_HB_), arm bending source (S_AB_), and knee bending source (S_KB_).

For each source, there is a range of voltage that can be generated according to the applied mechanical (motion) force and the frequency of the movement, in addition to the bending angle. The models for these sources are discussed later.

As previously presented, in the studied SpWBAN system model, there are microgrids, where each one consists of a group of sensors integrated with energy harvesting sources to self-power the sensors. Let us assume that we have a SpWBAN with only three sensors where each sensor adopts one of the three sources (SHB, SAE, and SKB). For example, the sensor near to the heart will depend on SHB, the sensor implemented on the arm elbow depends on SAB, and the sensor implemented on the leg knee depends on SKB.

In the following subsections, we analyze three performance metrics (energy consumption, network lifetime, and network throughput) to evaluate the efficiency of the operating SpWBAN.

**Energy Consumption Analysis**

Based on the MAC model presented in [28], the average energy consumption of every sensor in a SpWBAN, based on a data arrival rate λ and a service rate μ, is expressed as follows:

(3) $$E_{sensor}=E_{send}-E_{t,source}+E_{inactive}$$

We examine the model of a saturated SpWBAN where the sensors send data continuously to the collector without any inactivity period (i.e., $T_{inactive}=0$, $+E_{inactive}=0$ and $E_{t,source}=E_{p,\;source}$). Therefore:

(4) $$E_{sensor}=E_{send}-E_{t,source}$$

where $E_{sensor}$ represents the average energy consumption of a sensor, which collects readings and sends it with energy $E_{send}$, expressed by:

(5) $$E_{send}=E_{T-multi}+E_{R-multi}+E_{T-single}+E_{R-single}+E_{O}$$

where $E_{T-multi}\;and\;E_{R-multi}$ represent the energy consumed in transmitting and sending, respectively, in multi-beam mode, and $E_{T-single}\;and\;E_{R-single}$ represent the energy consumed in transmitting and sending, respectively, in single beam mode. As defined in [23], $E_{O}$ is the overhead energy consumption where the sensor becomes active and turns from a transmitting state to a receiving state and vice versa. We assume that sensors are always awake; and therefore $T_{wakeup}=0\;and\;E_{wakeup}=0$. Therefore:

(6) $$E_{O}=E_{turn}=n\times \left(P_{turn}\times 2T_{turn}\times k\right)$$

We define k as the number of data packets that are sent successfully by a sensor during the allocated period T, which is calculated as:

(7) $$k=\frac{\lambda T}{n_{packet,sensor}}$$

where $n_{packet,sensor}$ is the number of bits per sensor packet.

The total energy consumption of SpWBAN is the summation of energy consumed at the three sensor nodes:

(8) $$E_{sensor,\;total}=E_{sensor1}+E_{sennsor2}+E_{sensor3}=\;(E_{send1}-E_{t,S_{HB}})+(E_{send2}-E_{t,S_{AB}})+\left(E_{send3}-E_{t,S_{KB}}\right)$$

The average of SpWBAN for the energy consumption $E_{sensor,\;average}$ for the three sensors is calculated as:

(9) $$E_{sensor,\;average}=\frac{E_{sensor,\;total}}{3}$$

**Network Throughput**

We analyze the throughput generated from SpWBAN in the case that the sensor nodes are capable of sending data, are self-powered nodes and have data to send. We hypothesize that for at least 75% of the frame duration, self-powered sensor nodes can send. In contrast, the sensor nodes that have no self-powering capability have to wait for some time to save energy. Related to the MAC model presented in [28], the following formula is used to calculate the network throughput:

(10) $$Throughput=\frac{\lambda T_{active}}{T}$$

where $T_{active}$ is the active time when the sensor nodes are sending data.

**Network Lifetime**

We hypothesize that sensor nodes can last as long as there are body movements that can help in powering the nodes, as discussed previously. Accordingly, the lifetime of the network can be computed by considering the lifetime of the first dead sensor node. Considering the related MAC model presented in [28] and based on the energy consumed $E_{send}$ and the energy generated $E_{t,source}$ for that node during the allocated period T, the network life time can be prolonged by a ratio calculated from the following formula:

(11) $$T_{lifetime-ext}=\frac{E_{t,source}}{E_{send}}T$$

**D.**  **Smart Sensors Operation Management**

The collector or hop employs a lightweight machine learning (ML) algorithm and performs some analytics to reach a decision whether to continue accepting readings from a sensor or a suite of sensors or to direct them to minimize the active time or to stop sending readings. The collector will receive feedback from sensors about the generated level of harvested energy or voltage and other parameters, such as sensor location and monitored biometric priority. There are three sensor types, which are heart sensor, arm sensor, and knee sensor. There are also three levels of sensor priority, which are high, medium, and low. For example, if the collector receives data from a sensor referring to a low level of generated voltage and that sensor is monitoring a low-priority biometric, then, based on the employed ML algorithm, the collector makes a decision about continuing to receive readings from the sensor or applying some restrictions. If there is a very low level of generated voltage (leading to high energy consumption), the collector directs the sensor to stop activity until an acceptable level of the generated voltage is reached based on the operating requirements of the sensors.

**Figure 3:**
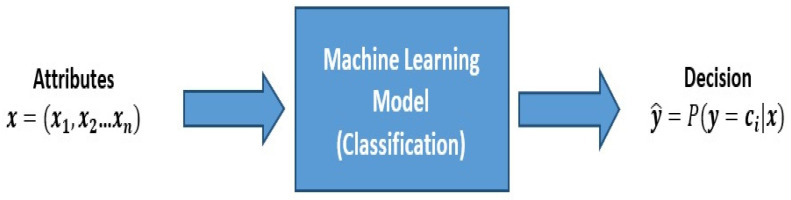
Machine learning-based management of health monitoring services allocation.

To model the implemented ML at the collector, mathematical analysis is implemented by utilizing machine learning algorithms for supervised classification. The collector depends on a training set of the acceptable and unacceptable operating ranges of the various implemented sensors. The collector reaches a suitable decision to either continue receiving **readings** from a sensor, to skip readings from that sensor or to allow sensors to operate with limited active time. Several classification algorithms are used, including decision trees, support vector machines, and neural networks. These algorithms can be used to test the possibility of enabling effective multiple health monitoring services. The machine learning models take in the collected preprocessed sensor-related data, which is classified into the most pivotal attributes according to the health monitoring service, and then it produces the decision on providing the monitoring services or not. Figure 3 shows a simple explanation for the machine learning model. Multiple attributes x, such as generated voltage level and sensor priority and location, are fed to the ML models, which were trained to reach decisions on providing a health monitoring service with or without restrictions or stopping service provision. We assume three classes ($c_{1}\;\mathrm{or}\;c_{2}\;or\;c_{3}$) where $c_{1}$ refers to continue working without restrictions (no changes on the active time), $c_{2}$ refers to continue working with restrictions (a change in the active time to be less), and $c_{3}$ refers to stop working.

**Figure 4:**
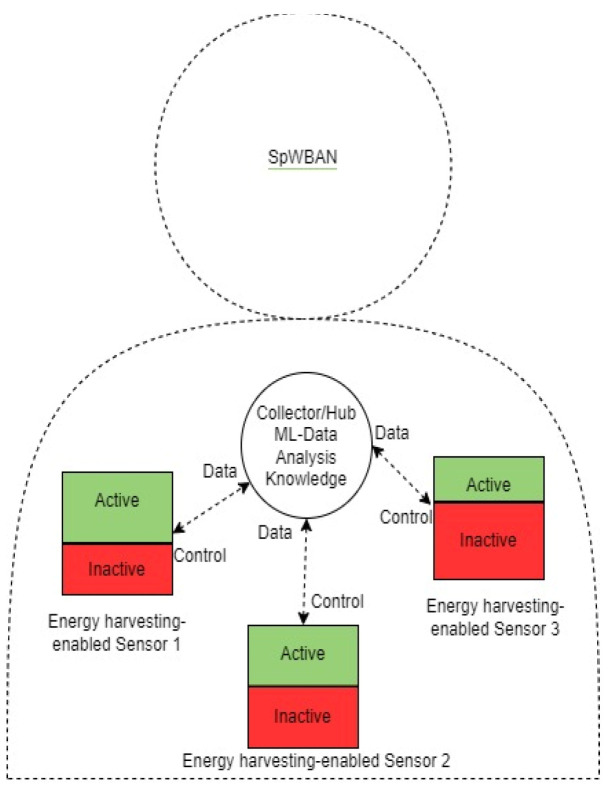
SpWBAN with smart machine learning-based operations.

Based on the decision taken by the collector, related sensor nodes are notified. For instance, in the case that a sensor node has a low generated voltage, which might affect the quality of service, the collector directs that sensor to stop working and enter inactive mode. This can be interpreted as having a decreased value or zero value for and of that node. Accordingly, this affects the throughput in the network and the overall lifetime. The following figure and algorithm pseudo code explain the SpWBAN design that applies a smart management system. Figure 4 illustrates a system model of SpWBAN, which employs machine learning at the main network controller and hub. All the sensors are approximately equal distance from the controller. Based on the analyzed data received from sensors, the controller may direct the sensors to increase or decrease the active time period. The collected sensor data include measurements, the level of generated voltage and harvested energy at each sensor and the amount of energy consumed in transmitting readings. Algorithm 1 introduces the main operations executed by the controller.
**Algorithm 1 Smart Management of SpWBAN Operations by the Controller****Input** SpWBAN Sensor Configuration Parameters $E_{T-multi},E_{R-multi},E_{T-single},E_{R-single},\;E_{O}$ Energy-related parameters initialization $E_{inactive}$ Sensors active time $T_{inactive}andT_{active}$**Operations** Run operating loops    Check energy levels at Sensors $E_{t,source}$   Generate voltage based on applied forces on nano-enriched piezoelectric materials V   Calculate the energy generated from the piezoelectric materials $E_{p,\;source}$   Adjust the data sending rate λ and number of data packets sent k   Compute the energy consumption for sending considering the harvested energy and sensor activity   Compute the total energy consumption in SpWBAN $E_{sensor,\;total}$   Estimate SpWBAN lifetime $T_{lifetime-ext}$   Estimate SpWBAN throughput Throughput   Recall SpWBAN from trained datasets and models    Check the energy level generated and consumed at each sensor based on trained models    Update the activity duration of sensors based on the classification of their energy levels**Output** SpWBAN sensors configuration   SpWBAN lifetime   SpWBAN throughput

**Figure 5:**
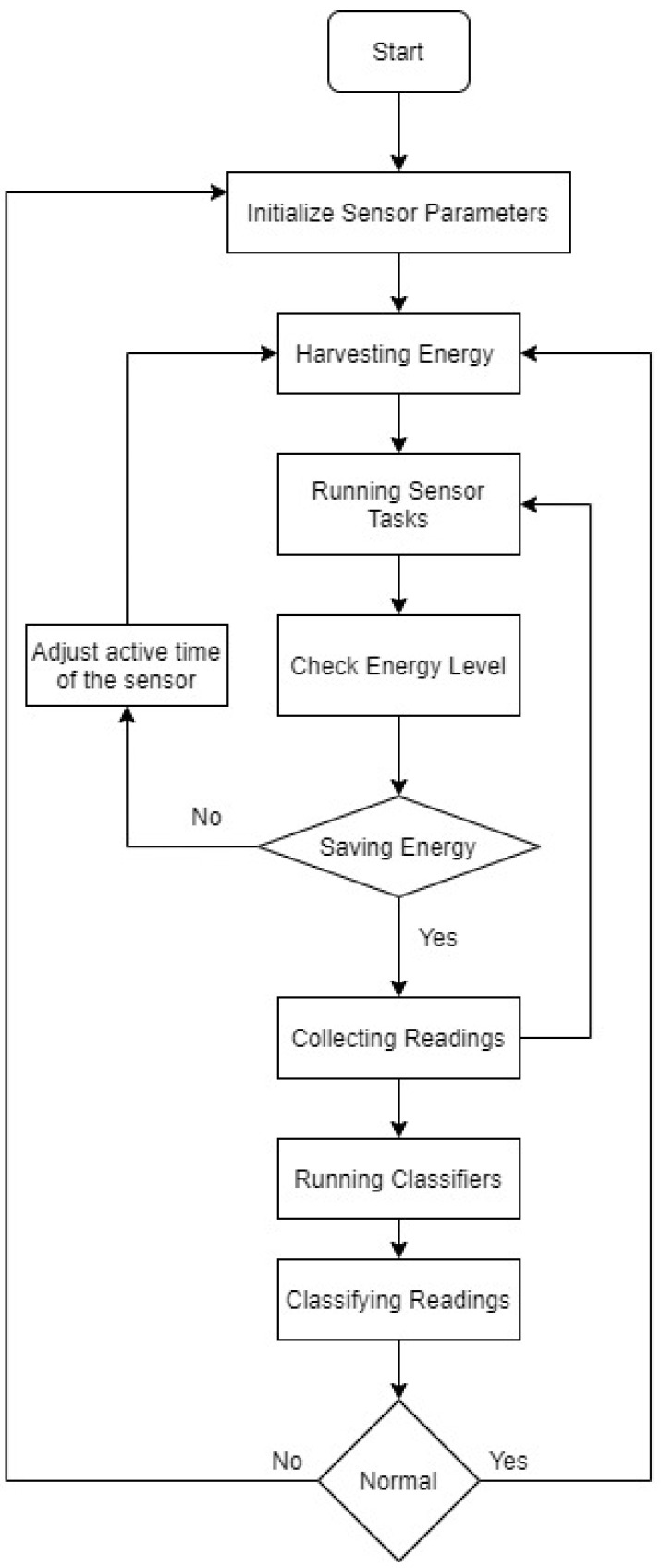
Flowchart of the main managed operations in SpWBAN.

Figure 5 illustrates a flowchart of the SpWBAN-managed operation where, based on the employed multi-class machine learning algorithms and the trained data, the controller can reach specific classes for reading from all sensors. Accordingly, the controller can direct the sensors to prolong or shorten the active duration.

## 4. Nano-Biosensor Design and Characterization

**Figure 6:**
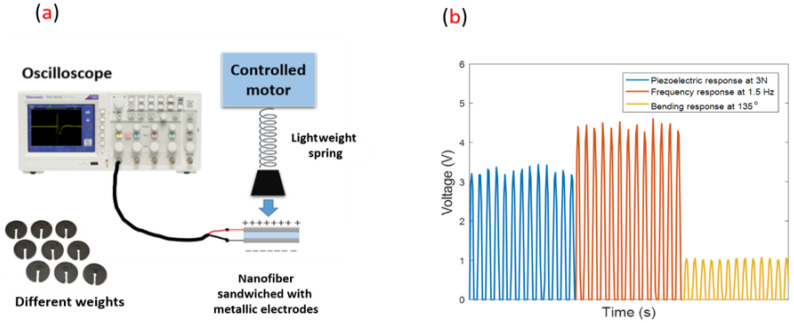
(**a**) Schematic of the piezoelectric characterization under applied force mechanical excitations. (**b**) The oscilloscope signals for the piezoelectric response at 3N, the frequency response at 1.5 Hz, and the bending response at 135°.

The proposed nano-biosensors consist of electrospun PVDF nanofibers mats fabricated as follows. First, 10 wt.% of PVDF (Kynar^®^, King of Prussia, PA, USA) polymer was dissolved in Dimethylformamide (DMF 98%, Sigma Aldrich, Taufkirchen, Germany) at room-temperature overnight with stirring. In the electrospinning process, the dissolved polymer was fed through a needle with a constant feed rate of 1 mL/h and applied to an electric high voltage power supply (Spellman, Hauppauge, NY, USA) up to 25 kV and then deposited on an electrically-grounded collector drum. The generated nanofibers mats were analyzed through a simple piezoelectric setup, as shown in Figure 6, where the output voltage was measured using a Tektronix MDO3014 oscilloscope under different applied mechanical vibrations.

**Figure 7:**
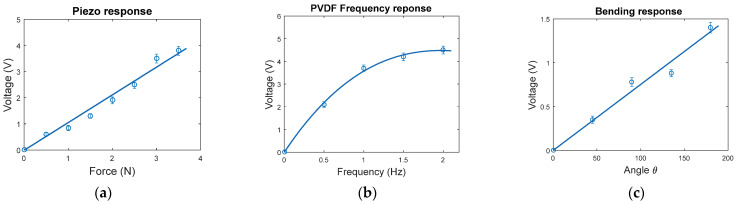
The Piezoelectric (**a**), frequency (**b**), and bending (**c**) response characterizations of the PVDF electrospun nanofibers.

The piezoelectric characterizations, including the piezoelectric, frequency, and bending responses, that are discussed in this section are shown in Figure 7. For the piezoelectric response, the output voltage increases linearly as long as the force increases at 1 Hz frequency, as shown in Figure 7a. When the force fixed at 3.5 N and the frequency is tuned, the output voltage increases and is saturated at 2 Hz with a voltage amplitude of 4.2 V, as shown in Figure 7b. The bending response in Figure 7c shows that the output voltage increases linearly as the angle increases.

## 5. Self-Powered WBAN Evaluation

We evaluated the developed SpWBAN model via a scenario created and tested using the Mathematica software. We assume one traffic type, which is sensed data and related readings collected by the sensors, however, with different arrival rates. In the model, the sensors are mounted at three different locations on the body. The sensor locations are the heart, elbow, and knee. In the studied scenario, we consider communication between the three sensors and the collector as one-hop communication with on body radio propagation. We evaluate the SpWBAN operation based on the energy consumption, average packet delay, and network lifetime. We compare the performance of the modeled SpWBAN with WBAN implementing the LEDA MAC protocol in [28]. The following table (Table 2) shows the simulation parameters.

**Figure 8:**
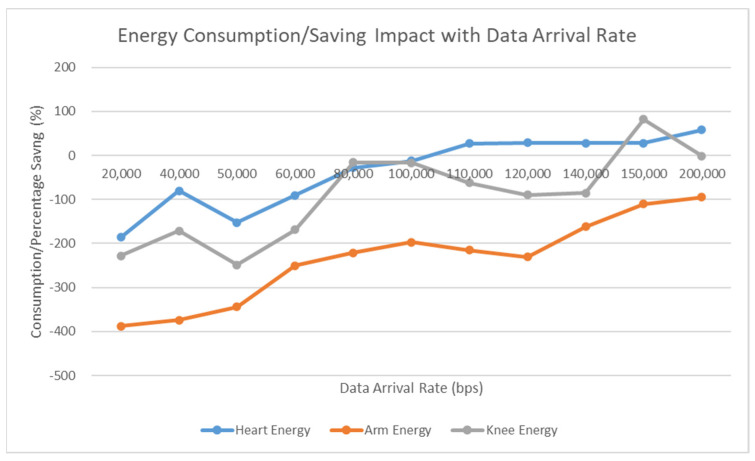
Energy consumption and saving with respect to varying data arrival rates.

We analyzed the energy consumption of SpWBAN versus the data arrival rate and the potential energy consumption and savings in the network. Figure 8 shows that operating SpWBAN with self-powering sensor nodes can optimize the energy consumption. Increasing the data arrival rates results in more energy consumption; however, with the self-powering sensors, the energy consumption effect is relieved. When varying the data arrival rates, the network is capable of saving a notable amount of energy that can support a sustainable long-term operation of the network. We compared the amount of energy consumed in case of running different sensors to support multiple levels of data rates. We found that the sensor mounted at the elbow generates much energy compared with the sensors mounted at the heart and knee. Accordingly, depending on the simulated scenario, the elbow sensor achieves average energy savings of 61% and 85% with respect to the sensors at the knee and heart, respectively.

We adopted three-layer neural network model as a multi-class machine learning algorithm where we generated a training data set for three sensors based on the generated energy from the implemented piezoelectrical material. The input layer takes three inputs: generated energy, source type, and level of generated energy (low or medium or high). There are three possible output classes: normal, moderate, and abnormal. Based on the operations presented in Figure 5, we classified the operation of each sensor and generated a notice about its current status while operation and testing.

**Figure 9:**
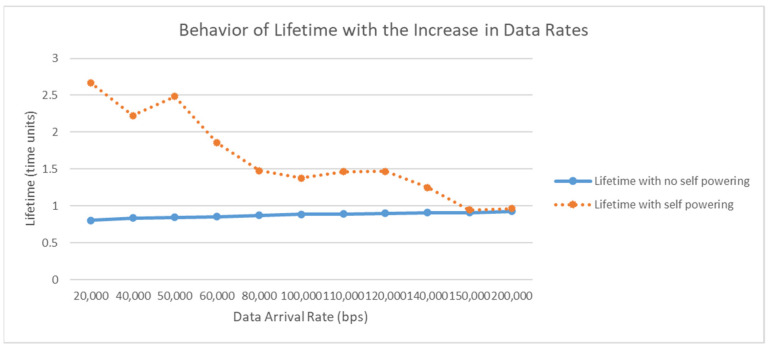
Improvement in network lifetime.

For the network lifetime, the sensors nodes are equipped with self-powering piezoelectric materials. Based on the expected movements, the generated energy and the consumed energy per sensor node, Figure 9 shows the improvement that can occur in the network lifetime when using the self-powered sensor nodes. The figure also provides the ratio of lifetime prolongation with respect to the data arrival rate. As expected, the lifetime of the network is affected negatively with increasing data arrival rates. However, adopting self-powering sensors leads to an increase in the lifetime of SpWBAN by 47% on average, depending on the operating data arrival rate.

**Figure 10:**
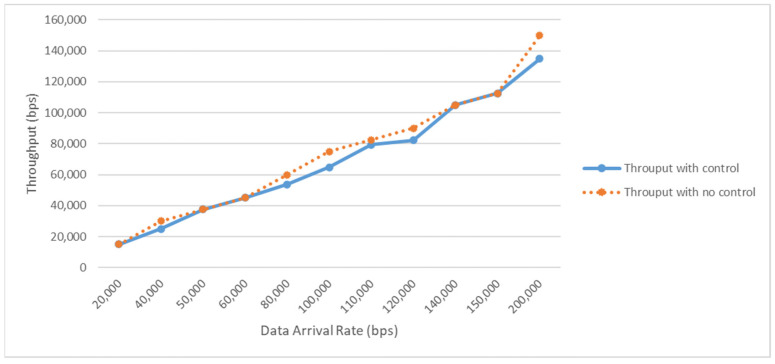
Network throughput.

Figure 10 shows the effect of having self-powering sensor nodes in SpWBAN and the ability of the sensors to be active most of the frame duration by adopting the single-beam transmission mode. We assume that during most of the frame duration, the sensor nodes can send data; however, if the harvested energy of each sensor does not meet the required operating level, the time allocated to each sensor node cannot exceed 75% of the frame duration. As can be observed in the figure, the applied machine learning management of control sensor operations and data sending results in good levels of network throughput when controlling the allocated time for sensors to send. Moreover, adjusting the activation time that the sensors send data has a significant impact on energy saving in the network, and hence the lifetime.

## 6. Conclusions

In this work, we have proposed a system model for self-powered WBAN (SpWBAN) to offer reliable health monitoring services. We developed fabricated PVDF nanofiber mats with specific electrical and physical characteristics that enable the design of piezoelectric nano-biosensors. The adopted SpWBAN model depends on the energy harvesting-based MAC protocol to achieve energy-efficient data transmission and to prolong the network lifetime. We applied some modifications in the MAC protocol to enable machine learning-based control where sensors enter dynamic active time slots based on their activity and the classification for the harvested energy by the implemented nano-enriched piezoelectric materials. Our evaluation of the SpWBAN showed a significant amount of energy saving based on using three self-powered sensors at three positions on the human body. Additionally, the analytical results yielded an improvement in the network lifetime when applying the energy harvesting system. In addition, controlling the operation of sensors based on machine learning-based management enables acceptable network throughput ranges while achieving energy saving and prolonging the network lifetime.

Our future work will focus on developing a SpWBAN system model with a hierarchical network architecture of multiple microgrids with different types of self-powered nano-biosensors under various operating conditions. Additionally, we will develop software-defined networking to enable reliable data routing and forwarding among microgrids and the main controller and the Internet. Our future work will also involve developing more sustainable and biodegradable nano-biosensors.

## Figures and Tables

**Table 1:** The fitting equations for the different applied forces, frequencies, and bending angles.

The Voltage Generated Based on:	Equation
The applied force F(V_force_):	V_force_ = 1.0603× F − 0.012263
The movement or signal frequency f(V_freq_):	V_freq_ = 0.18607 × f^3^ − 1.8891 × f^2^ + 5.2735 × f−0.00083565
A bending angle θ(V_angle_):	V_angle_ = 0.0075111 × θ + 0.001

**Table 2:** Simulation parameters.

Parameter	Value
Multi-mode transmit power	0.5 × 10^−3^ Watt
Magnetic flux density, Magnetic induction	1 G → 10^−4^ T = 10^−4^ Wb/m^2^
Multi-mode receive power	1 × 10^−3^ Watt
Single-mode transmit power	3.162 × 10^−6^ Watt
Single-mode receive power	6.31 × 10^−6^ Watt
DRF time	0.1235 ms.
Beacon time	0.1235 ms.
Notification time	0.1235 ms.
Turn time	0.1235 ms.
Data time	2.3 ms.
Frame time	1 s.
Operating frequency	0.5 Hz to 5.8 Hz
Generated voltage	0.5 V~4 V
Conductive material resistance	$15\times 10^{3}\Omega$
Piezoelectric material capacitance	5 nF
Energy generation time	1.5 × 10 ms.
Number of bits/packet	2233 bits
Ack time	0.06 × 10 ms.
Inactive time	0
Turn power	1 × 10^−6^ Watt

## Data Availability

Not applicable.
